# Role of Myokines on the Bone Metabolism of Craniofacial Region: A Scoping Review

**DOI:** 10.3390/dj13090400

**Published:** 2025-08-31

**Authors:** Ahana S. Rajan, Eiji Tanaka

**Affiliations:** 1 Department of Orthodontics, Meenakshi Ammal Dental College and Hospital, Meenakshi Academy of Higher Education and Research (Deemed to be University), Chennai 600095, Tamil Nadu, India; drahana.ortho@madch.edu.in; 2 Department of Orthodontics and Dentofacial Orthopedics, Tokushima University Graduate School of Biomedical Sciences, Tokushima 770-8503, Japan

**Keywords:** bone metabolism, craniofacial region, muscle-bone crosstalk, myokine

## Abstract

**Background:** The craniofacial region is functionally unique, with close interaction between muscles and bones during mastication, speech, and facial expression. Although myokines, muscle-derived signaling molecules, are increasingly being studied in relation to bone metabolism, most studies have focused on limb muscles and long bones. Given the developmental and functional specificity of craniofacial structures, this article aims to map the current evidence on myokines involved in craniofacial bone metabolism and to identify gaps in order to guide future research. **Methods**: We conducted a literature search in PubMed and Scopus (January 2000–July 2025), combining both free-text keywords and MeSH terms to ensure comprehensive retrieval of relevant articles. **Results:** Nine articles from the extensive search were included in this review that adhered to the eligibility criteria. The myokines that were reported include interleukin-6, insulin like growth factor-1, and myostatin and irisin. **Conclusions:** Further research is required into the mechanism by which craniofacial muscle-derived myokines regulate local bone metabolism, as this knowledge could pave the way for novel therapeutic strategies that leverage myokine signaling, which could be applied in the context of orthodontic and orthognathic treatments, maxillofacial reconstruction, or age-related bone loss.

## 1. Introduction

Since the craniofacial region is continuously remodeled, its dynamic nature is influenced by the complex interplay between genetic, mechanical, and biochemical factors [1]. In this craniofacial region, the bones and muscles originate from the neural crest cells and the mesoderm, respectively, during their embryonic development [2]. Muscle contractions begin as early as 6th–8th week of intrauterine life, and their absence can have a profound impact on the craniofacial architecture [3]. Postnatally, the masticatory muscles impact the growth patterns of the maxilla and mandible by exerting mechanical forces to regulate remodeling, which in turn dictates the sutural growth, apposition, and facial dimensions [4]. Synchronized development of these tissues is essential for normal structure [1].

The human masticatory system is a complex musculoskeletal system in which the masticatory muscles stretch and compress in the temporomandibular joint (TMJ), and jaw movements interact [5]. If one of these factors alters, it influences the others. This particularly concerns the activation patterns of the jaw muscles, which adapt more or less easily to a change. Over the last few decades, the mechanism of muscle-bone crosstalk has been investigated. This comprises the loads generated during muscle contraction as well as a biochemical component involving soluble molecules [6]. The close association between muscles and bones is regulated by various biochemical pathways, and even the slightest disturbance can disrupt the cellular cascade [7]. At the molecular level, the masticatory muscles respond to the mechanical stimuli by secreting myokines. These molecules permeate through different barriers to transfer the stimuli and maintain homeostasis, thereby demonstrating their autocrine, paracrine, and endocrine nature [6].

Myokines, produced by muscles during physical activity, may exert anti-inflammatory and anti-tumor effects by directly influencing the tumor microenvironment and the immune system [8]. These signaling molecules have the potential to modulate tumor cell growth and viability, suggesting that exercise may contribute to cancer prevention and control through complex biochemical mechanisms [9]. Furthermore, myokines are considered as the mediators of the balance between the pro-inflammatory and anti-inflammatory responses [10]. This highlights the importance of muscle function in determining good health and preventing diseases. The following molecules have been defined as myokines in the recent literature: interleukin (IL)-5,6,7,8,15; brain-derived neurotrophic factor (BDNF); irisin; insulin-like growth factor (IGF); β-aminoisobutyric acid (BAIBA); matrix metalloproteinase (MMP)-2; and fibroblast growth factor (FGF)-2 [11,12].

Despite growing evidence of muscle-bone biochemical interaction, the precise functions of myokines in cranial bone metabolism remain poorly understood. Most studies have focused on how these molecules affect long bones, like those in the limbs. The craniofacial skeleton is different, featuring unique developmental origins, biomechanical demands (mastication, speech, and facial expression), and muscular attachments. Because of this, we cannot simply apply findings from limb bone studies to the craniofacial context.

Importantly, there is no existing review that specifically investigates how craniofacial muscles communicate with bone via myokines—a major knowledge gap given the craniofacial region’s importance in orthodontics, facial surgery, and age-related bone changes. Thus, the primary aim of this scoping review was to map the current evidence on myokine-mediated signaling in craniofacial bone metabolism.

Furthermore, understanding this relationship is crucial because myokines could affect orthodontic treatments, developmental issues, and craniofacial rehabilitation techniques. The second aim of this scoping review was to summarize the evidence thus far on the role of myokines in craniofacial bone metabolism and identify gaps to guide future research.

## 2. Materials and Methods

This scoping review was performed based on the updated recommendations of the Joanna Briggs Institute [13]. The results are reported in accordance with the Extension for Scoping Reviews of the Preferred Reporting Items for Systematic Reviews and Meta analyses statement (PRISMA-ScR) [14]. In line with best practices for scoping reviews, we pre-registered our protocol on the Open Science Framework (OSF) before initiating the review. The preregistration includes our research questions, inclusion/exclusion criteria, search strategy, and data charting plan, thereby promoting methodological transparency and reducing reporting bias. The link of the OSF is as follows: https://osf.io/4j3k9 (accessed on 26 August 2025).

### 2.1. Search Strategy

A methodical literature search was conducted in PubMed and Scopus databases from January 2000 to July 2025 to procure relevant articles. The keywords used were myokines, bone metabolism, and craniofacial region. Table 1 represents the search terms, mesh words, and Boolean logic used for PubMed and Scopus databases.

### 2.2. Eligibility Criteria

Based on the PICO framework, the eligibility criteria for the study selection was divided into Population, Intervention, Control, and Outcome (Table 2). To guide our scoping review, we structured our main research question using the PICO framework as ”In models involving craniofacial bones (P), does exposure to muscle-derived myokines or their modulation (I), compared to conditions without myokine exposure or differing muscle activity patterns (C), influence key bone metabolism outcomes such as remodeling rates, structural integrity, density, or molecular markers (O)?”

### 2.3. Study Selection

**Figure 1:**
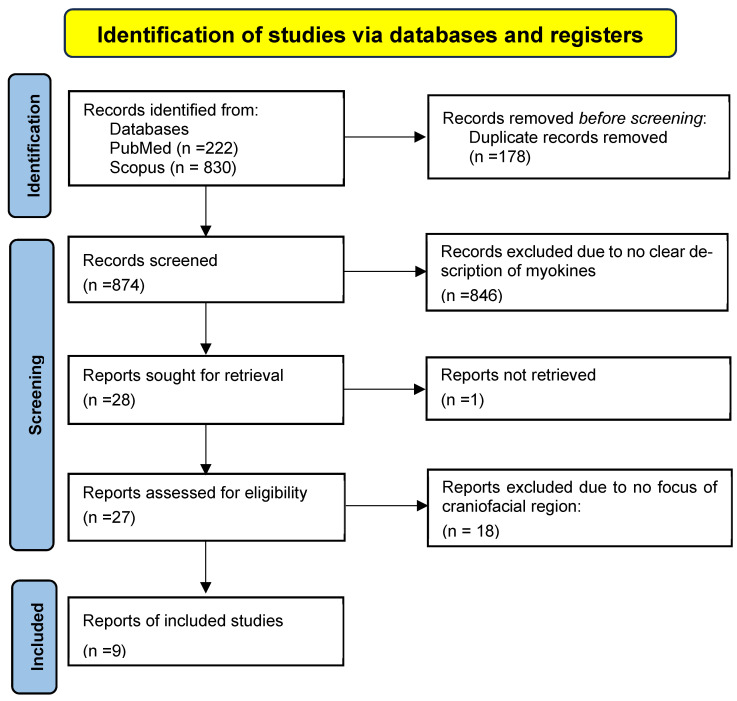
PRISMA Flowchart.

This review was performed by three independent reviewers to ensure transparency and methodological rigor. The inclusion criteria encompassed original articles and review articles that had a clear description of myokines and focused on the craniofacial regions. We excluded studies that did not involve myokines in the craniofacial regions as well as conference papers and dissertations. After an initial search using the keywords, relevant articles were filtered based on the abstracts and title. Eventually, selected articles were retrieved according to the eligibility criteria assessed for their full-text potential using the Rayyan^®^ application (Copyright© 2025 Rayyan) [15]. Figure 1 shows the PRISMA flowchart that was used to document the search results and study selection process. Any disagreement was resolved by discussion by the two authors.

## 3. Results

### 3.1. Search Results

A total of 874 articles were screened from the two databases after eliminating the duplicates. Of these, 846 articles were excluded as they had no clear description of myokines. Twenty-eight articles were assessed in full text, and nine articles that met the eligibility criteria were finally included in the review. A summary of the included articles is shown in Table 3. The quality of the included articles was not assessed.

### 3.2. Role of Myokines in Craniofacial Region

The craniofacial system, especially the masticatory apparatus, works in a well-regulated manner in response to constant functional and mechanical demands through remodeling [25]. The major bones, such as the mandible, maxilla, and temporal bone, form a dynamic structural framework that houses the TMJs. Meanwhile, muscles such as the masseter, temporalis, and medial and lateral pterygoids are responsible for complex movements such as chewing, speaking, swallowing, and facial expressions [26].

Although mechanical load is the key instigator of bone adaptation through mechanotransduction [27], emerging evidence suggests that skeletal muscles also play a biochemical role in bone metabolism by secreting myokines. These signaling peptides and cytokines are produced and released by muscle fibers in response to contraction and other stimuli and can exert autocrine, paracrine, and endocrine effects [28]. Myokines have been implicated in a wide array of physiological processes, including metabolism, inflammation, and tissue regeneration, and have attracted considerable interest in the context of muscle - bone crosstalk [6,11,29,30].

Myokines have been shown to directly affect bone cells [31,32,33]. For example, irisin, a cleavage product of fibronectin type III domain-containing protein 5 (FNDC5), has been shown to enhance osteoblast differentiation, inhibit osteoclast activity, and increase cortical bone mass in long bones [27,29,30,34]. Myostatin, a member of the transforming growth factor (TGF)-β superfamily, acts as a negative regulator of muscle growth and also inhibits bone formation by suppressing osteoblast proliferation and stimulating osteoclastogenesis [35,36,37,38]. IL-6, another well-characterized myokine, exerts dual effects depending on the context. It contributes to both bone resorption and bone formation under different conditions [15,39,40,41]. IL-6 is released from skeletal muscles, and its plasma concentration was found to increase after physical exercise [42]. FGF21 and BAIBA are additional myokines involved in bone – fat - muscle cross-communication [43]. IGF-1 is released from skeletal muscles, although it is primarily produced in the liver [44]. These myokines are expressed by osteocytes and increase with mechanical loading, playing a key role in the interplay between bone and muscle [45,46].

To date, the majority of studies have focused on limb muscles (e.g., the gastrocnemius and the quadriceps) and long bones (e.g., the femur and the tibia). These studies have used models of exercise, mechanical loading, or genetic modifications to explore the impact of muscle-derived signals on skeletal homeostasis [29]. In contrast, few studies have investigated craniofacial muscles, particularly the masticatory muscles, as sources of bone-regulatory myokines. This is surprising, given the functional importance of these muscles and their distinct developmental origin. Unlike limb muscles, which originate from somites, masticatory muscles develop from cranial paraxial mesoderm and neural crest-derived mesenchyme, specifically from the first pharyngeal (branchial) arch [47]. Their innervation (via the mandibular branch of the trigeminal nerve), vascularization, and embryological regulation differ markedly from those of the trunk and limb musculature. Craniofacial bones also arise from distinct developmental pathways, largely through intramembranous ossification, as opposed to the endochondral ossification observed in long bones [48]. These developmental, anatomical, and functional differences suggest that muscle-to-bone signaling in the craniofacial region may involve unique regulatory molecules and mechanisms.

#### 3.2.1. IL-6

Yamamoto et al. [16] investigated the effect of mechanical stress mimicking occlusal force on the cytokine expression in mandible-derived osteoblast cells. They found that the application of hydrostatic pressure increased the production of IL-6 and TNF-α production through p38 MAPK pathway (mitogen-activated protein kinase pathway). Shinoda et al. [17] evaluated IL-6 expression in the synovial fluids of healthy individuals and TMD patients. They indicated that IL-6 levels were higher in TMD patients with osseous changes, suggesting its role in TMD pathogenesis.

Pereira et al. [18] evaluated the effect of physical training on the orthodontic tooth movement in mice. They demonstrated that both the aerobic and resistance training groups exhibited enhanced maxillary bone quality, with increased bone mineral density and bone trabecular volume and reduced IL-6 levels. However, the orthodontic tooth movement was significantly reduced in the trained group, which had more osteoblasts and fewer osteoclasts.

#### 3.2.2. Myostatin

Ravosa et al. [19] used “Mighty Mouse” (myostatin-deficient) and diet-manipulated rabbit models to assess the effect of increased masticatory load on craniofacial bone and cartilage. They found that the hypertrophied muscles impacted the bone mineral density of the TMJ and mandible due to the altered bite force transmission. Myostatin-deficient mice had lower TMJ bone mineral density. In another experiment, Nicholson et al. [20] found that the biomineralization of the subcondylar cartilage was much lower in myostatin-deficient mice than in wild mice when subjected to an increased masticatory load. Byron et al. [21] evaluated the cranial sutural complexity and muscle mass of myostatin-deficient mice and indicated that increased temporalis muscle mass reduced the sagittal suture connective tissue stiffness, which could affect the distribution of functional load.

#### 3.2.3. Irisin

Yang et al. investigated the presence of irisin in the periodontal and pulpal tissues of Sprague–Dawley rats [22]. They found that the irisin was actively expressed in these tissues, with increased osteoblast production observed in pulpal cells following irisin injection. The expression of irisin varied in different tissues, suggesting cell-type-specific responses. In another study, Yang et al. [23] explored the expression and regulation of irisin in Wistar rats alongside orthodontic tooth movement. Orthodontics tooth movement was hampered on the compression side due to the osteogenic effect of irisin on periodontal ligament cells.

#### 3.2.4. IGF-1

Liu et al. evaluated the level of IGF-1 in Wistar rats with unilateral loading of the TMJ with occlusal splints [24]. They found that degenerative changes with IGF-1 overexpression were present in the condylar and subcondylar tissues on the non-chewing side, suggesting a role for IGF-1 in osteogenesis.

## 4. Discussion

### 4.1. Summary of Key Findings

This scoping review identified nine studies examining the role of specific myokines—namely IL-6, IGF-1, myostatin, and irisin in regulating craniofacial bone metabolism. The evidence suggests that myokines can exert both pro- and anti-osteogenic effects depending on the context, such as the type of mechanical stimulus, the tissue involved, and the disease state. IL-6 was the most frequently studied myokine, with research showing its elevated expression in temporomandibular disorders (TMD) and in osteoblasts subjected to simulated occlusal stress. Myostatin, which is primarily known for inhibiting muscle growth, was shown to impact bone mineral density and cranial suture mechanics in models of increased masticatory load. Irisin and IGF-1 were associated with enhanced osteogenic responses in periodontal tissues and TMJ cartilage, respectively. While these findings are promising, the literature is sparse and heterogeneous, highlighting an important yet underexplored field of craniofacial bone biology.

### 4.2. Comparison with Myokine Research in Other Skeletal Regions

Most existing myokine research has focused on long bones and limb musculature. Studies on limb muscles have demonstrated that myokines such as IL-6 and irisin are released in response to exercise, contributing to improved bone density and turnover, largely through systemic effects [28,31,34]. In contrast, craniofacial bones, which undergo continuous remodeling due to localized forces from mastication and speech and are therefore exposed to region-specific myokines that may act in a more paracrine or autocrine manner. For instance, irisin has been shown to reduce orthodontic tooth movement by enhancing osteogenesis in the maxilla, an effect that has not been observed in long bones [23]. Similarly, IGF-1 responses in the TMJ under unilateral load differ from the adaptive responses seen in weight-bearing joints such as the knee or hip [24]. These differences highlight the importance of studying myokine regulation in a craniofacial-specific context.

### 4.3. Functional and Developmental Uniqueness of Craniofacial Muscles and Bones

The craniofacial bones and muscles are developmentally and functionally distinct from their axial and appendicular counterparts. Masticatory muscles, such as the masseter and temporalis, arise from the cranial paraxial mesoderm and neural crest cells. In contrast, limb muscles originate from somites [47]. Similarly, craniofacial bones develop largely through intramembranous ossification, in contrast to the endochondral ossification of long bones [48]. These embryonic differences are reflected in function: craniofacial structures undergo frequent, low-amplitude loading during chewing and speech, whereas limb bones undergo intermittent, high-magnitude loading during locomotion. This unique environment may influence the type and amount of myokines secreted and how they interact with local bone cells. For instance, changes in masticatory muscle activity due to diet, parafunction, or muscle hypertrophy have been shown to modulate myokine expression and alter the bone microarchitecture in the TMJ and sutures [19,20,21].

### 4.4. Potential Mechanisms of Myokine Action in the Craniofacial Region

The interaction between muscle-derived myokines and craniofacial bone cells appears to be mediated by several pathways. IL-6, for instance, is upregulated in osteoblasts exposed to mechanical pressure as well as in the synovial fluid of TMD patients with osseous changes. IL-6 has been shown to influence osteoclastogenesis via the RANKL/OPG axis and the p38 MAPK signaling pathway, suggesting a role in both inflammatory and remodeling processes [16,17]. Irisin, a cleavage product of FNDC5, has osteoinductive effects in the periodontal ligament, promoting osteoblast differentiation and bone formation in areas subjected to orthodontic forces [22,23]. IGF-1 is believed to act as a key mediator of anabolic responses in cartilage and subchondral bone, especially under altered functional loading conditions such as those seen in the TMJ during unilateral mastication [24]. In contrast, myostatin acts as a negative regulator, reducing osteoblast proliferation and enhancing bone resorption, which may be particularly relevant in the context of hyperactive masticatory muscles [19,20,21].

### 4.5. Clinical Implications

Understanding the role of myokines in the craniofacial region could lead to new clinical applications. In orthodontics, for example, modulating myokine activity could influence the rate of tooth movement or help maintain bone integrity during prolonged treatment. For instance, the osteogenic effect of irisin cold be utilized to stabilize anchorage units or prevent root resorption. In TMDs, targeting IL-6 pathways may reduce inflammatory bone resorption and offer therapeutic benefits. Furthermore, knowledge of region-specific myokines could inform regenerative strategies in maxillofacial surgery, particularly in cases of trauma, reconstructive surgery, or age-related bone loss. In conditions such as idiopathic condylar resorption or craniofacial dysostoses, characterizing myokine expression could provide diagnostic or prognostic biomarkers.

### 4.6. Limitations

This scoping review has a few important limitations that should be acknowledged. First, the number of eligible studies was relatively small, with most relying on animal or in vitro models. While these provide valuable mechanistic insights, they limit the direct translation of findings to human craniofacial bone biology. Second, the included studies were heterogeneous in terms of experimental design, myokines investigated, and out-come measures. This diversity made it difficult to compare results directly or draw firm conclusions on the relative contribution of each myokine. Third, we did not conduct a formal risk of bias or quality appraisal, as this falls outside the scope of scoping reviews; however, this means that the strength of evidence cannot be graded. To address this, we implemented a study selection process involving three independent reviewers, working through titles, abstracts, and full texts to ensure consistency. Discrepancies were resolved through discussion, enhancing the reliability of our inclusion decisions. While we did not compute inter-rater agreement statistics (e.g., Cohen’s kappa), this consensus-driven approach is a well-recognized method to reduce selection bias and reinforce methodological rigor. Finally, although our search strategy was comprehensive, we did not systematically search the grey literature, and relevant unpublished studies may have been missed. Together, these factors suggest that while the current review provides a broad overview of existing knowledge, more standardized and well-designed human studies are needed to clarify the role of myokines in craniofacial bone metabolism.

## 5. Conclusions

Despite the growing recognition of the role of myokines in systemic musculoskeletal health, our scoping review reveals that research into the myokine-mediated regulation of craniofacial bone metabolism is still in its infancy. This disparity in research emphasis raises a significant knowledge gap. A better understanding of the specific myokine profiles of craniofacial muscles could provide new insights into the impact of mechanical loading, muscle activity, and inflammation on local bone remodeling, particularly in pathological conditions such as idiopathic condylar resorption, TMJ osteoarthritis, and craniofacial deformities. Furthermore, this knowledge could pave the way for novel therapeutic strategies that utilize myokine signaling to modulate bone homeostasis or regeneration in the craniofacial region. This could be applied in the context of orthodontic and orthognathic treatments, maxillofacial reconstruction, or age-related bone loss.

In summary, while the role of myokines in the limb muscle - bone interaction is now well understood, their function in the craniofacial muscle - bone crosstalk is a new but promising area of research. The unique structure and function of the masticatory system highlight the importance of region-specific investigations. Future studies should prioritize characterizing the secretome of craniofacial muscles, elucidating myokine-mediated signaling pathways, and evaluating their impact on bone cells and craniofacial morphology. Further research should go beyond the currently studied IL-6, IGF-1, myostatin, and irisin and explore their roles in areas like sutures, alveolar bone, and dental tissues. Addressing these knowledge gaps will be crucial to advancing our understanding of muscle - bone biology in the craniofacial domain and create new clinical strategies—for example, enhancing orthodontic anchorage with irisin modulation or mitigating TMD-associated bone resorption by targeting IL-6—that could bridge basic discoveries with patient care.

## Figures and Tables

**Table 1:** List of search words, mesh terms, and Boolean logic.

Concept 1: Myokines	Concept 2: Bone Metabolism	Concept 3: Craniofacial/Masticatory
myokine *	“bone remodeling”	craniofacial
“muscle-derived cytokine *”	“bone metabolism”	mandible OR mandibular
irisin	osteogenesis	maxilla OR maxillary
myostatin	osteoblast *	“temporomandibular joint” OR TMJ
interleukin-6 OR IL-6	osteoclast *	“facial bone *”
Cytokines [Mesh]	“bone mineral density”	“jaw bone *”
FNDC5 protein, human [Mesh]	“bone resorption”	“masticatory muscle *”
Myostatin [Mesh]	Bone Remodeling [Mesh]	masseter OR temporalis
Interleukin-6 [Mesh]	Osteogenesis [Mesh]	Mandible [Mesh]
	Osteoblasts [Mesh]	Maxilla [Mesh]
	Bone Resorption [Mesh]	Temporomandibular Joint [Mesh]

* indicates major topics of this review.

**Table 2:** PICO framework for eligibility criteria.

Population	Humans or animals (in vivo or in vitro studies) Craniofacial bones (e.g., mandible, maxilla, alveolar bone, temporomandibular joint, and cranial bones) Muscle-bone crosstalk in the craniofacial region
Intervention/exposure	Presence, secretion, or modulation of myokines. Muscle activity, injury, or stimulation affecting myokine release
Control	With vs. without myokine exposure Healthy vs. disease states. Different levels or types of muscle activity Craniofacial vs. non-craniofacial bones
Outcome	Bone metabolism indicators (bone formation, resorption, and remodeling) Changes in bone density, structure, or healing Molecular markers of bone turnover Histological or radiographic bone changes

**Table 3:** Studies selected describing myokines involved in craniofacial bone metabolism.

Author and Year	Population	Intervention	Control	Myokine Considered	Key Findings
Yamamoto et al. [16]	Mandiblederived osteoblast cells from 4–6-weeks old male mice.	Intermittent hydrostatic pressure (magnitude: 0.1, 1, and 6 MPa; time: 10, 30, and 60 min; frequency: 1 Hz)	Varying level of hydrostatic pressure.	IL-6	The hydrostatic pressure simulating the occlusal force influenced the RANKL/OPG ratio in maintaining the bone homeostasis through osteoclastogenesis.
Shinoda et al. [17]	48 patients with TMD	Synovial fluids from TMD patients with osseous changes.	18 healthy individuals	IL-6	IL-6 was increased in the patients with osseous changes due to chronic TMD and absent in healthy controls, suggesting its role in TMD pathogenesis.
Periera et al. [18]	Mice	Aerobic or resistant physical training combined with orthodontic tooth movement	Sedentary mice	IL-6, IGF-1	Increased BMD, BV, BV/TV, and IGF-1 production was noted in the maxilla in the physical training group.
Ravosa et al. [19]	Myostatin deficient and wild mice	Masticatory overloading through diet.	New Zealand white rabbit.	Myostatin	Tissue mineral density was lowest at the TMJ and highest at the symphysis.
Nicholson et al. [20]	Myostatin-knockout mice.	Increased jaw adductor activity due to masticatory overload	Wild-type mice	Myostatin	There was altered mineralization of condylar subchondral bone due to increased masticatory stress in myostatin-deficient mice.
Byron et al. [21]	Myostatin-deficient mice	Increased bite force due to hard diet and temporalis muscle mass	Wild-type mice	Myostatin	The sagittal suture connective tissue was less stiff due to hyper muscular temporalis and dissipated bite force better.
Yang et al. [22]	Sprague -Dawley rats	Presence of irisin in PDL, dental pulp, and osteoblasts through cell culture	Cultured human PDL, dental pulp, and osteoblasts	Irisin	Irisin presence in the rat and human PDL was confirmed. The irisin production was affected during osteoblast like differentiation.
Yang et al., 2023 [23]	Wistar rats; right side of the maxilla	Irisin injected submucosally along with mesial movement of molar	Wistar rats; left side of maxilla	Irisin	Osteogenic potential of periodontal ligament was enhanced, and it reduced the orthodontic tooth movement on the compression side.
Liu et al. [24]	Wistar rats	Unilateral loading with dental splints	Control-group rats	IGF-1	IGF-1 was increased in the non-chewing side, and condylar degeneration was seen on the chewing side.

## Abbreviations

The following abbreviations are used in this manuscript:

MPa	Mega Pascal
Hz	Hertz
RANKL	Receptor activator of nuclear factor kappa-Β ligand
OPG	Osteopontegrin
TMD	Temporomandibular disorders
TMJ	Temporomandibular joint
IGF-1	Insukin-like growth factor-1
IL-6	Interleukin 6
BMD	Bone mineral density
BV	Bone volume
TV	Trabecular Volume
PDL	Periodontal ligament
BNDF	Brain-derived neurotrophic factor
BAIBA	β-aminoisobutyric acid
MMP	Matrix metalloproteinase
FGF	Fibroblast growth factor
PRISMA-ScR	The Extension for Scoping Reviews of the Preferred Reporting Items for Systematic Reviews and Meta analyses statement
FNDC5	Fibronectin type III domain-containing protein 5
PICO	Population Intervention Control Outcome
MAPK	Mitogen activated protein kinase pathway
